# Implementation gaps in contemporary heart failure therapy: a two-country nationwide analysis

**DOI:** 10.1136/openhrt-2026-004245

**Published:** 2026-10-16

**Authors:** Anna Norhammar, Kari Anne Sveen, Johan Bodegård, Marcus Thuresson, Trond G Jenssen, Kåre I Birkeland

**Affiliations:** 1 Cardiology Unit, Department of Medicine, Karolinska Institute, Stockholm, Stockholm County, Sweden; 2 Capio St Görans Hospital, Stockholm, Sweden; 3 Department of Endocrinology, Morbid Obesity, and Preventative Medicine, Oslo University Hospital, Oslo, Norway; 4 RWE Research, Oslo, Norway; 5 Statisticon, Uppsala, Sweden; 6 Nephrology section, Department of Transplantation Medicine, Oslo University Hospital, Oslo, Norway; 7 Institute of Clinical Medicine, University of Oslo, Oslo, Norway

**Keywords:** HEART FAILURE, EPIDEMIOLOGY, Medication Adherence, Quality of Health Care, Drug Monitoring

## Abstract

**Background:**

To assess implementation of contemporary guideline-directed medical therapy (GDMT) for heart failure (HF) across two nationwide public healthcare systems, including treatment persistence across care settings and uptake of sodium–glucose cotransporter-2 inhibitors (SGLT2i).

**Methods:**

Data were obtained from the Patient, Prescribed Drug and Cause of Death registers in Norway and Sweden. Dose titration and discontinuation of newly initiated GDMT were evaluated in patients with an incident HF visit to *outpatient* or *inpatient care* between 1 January 2022 and 31 December 2022. Additionally, uptake of SGLT2i between 1 January 2020 and 31 December 2023, was assessed in patients with incident HF or prevalent HF.

**Results:**

During 2022, across Norway and Sweden, 57 779 patients initiated GDMT for HF in outpatient care and 54 239 patients in *inpatient care* with similar characteristics across the two countries. More than 80% of patients treated with SGLT2i remained on the target dose during follow-up; compared with approximately 5% and 40% for angiotensin receptor blockers and angiotensin receptor-neprilysin inhibitors, respectively. Discontinuation rates were consistently higher with GDMT initiated in outpatient care than with that initiated in *inpatient care*. Uptake of SGLT2i increased rapidly among patients with incident HF, reaching approximately 45% in Norway (2022) and 55% in Sweden (2023) whereas uptake among patients with prevalent HF remained low (10–12%).

**Conclusions:**

Implementation gaps in contemporary HF therapy persist. While uptake of SGLT2i increased rapidly following incident HF, utilisation among patients with established HF remained limited and treatment persistence was lower with GDMT initiated in outpatient care.

**WHAT IS ALREADY KNOWN ON THIS TOPIC**

- Guideline-directed medical therapy (GDMT) for heart failure is underused and often not titrated to target doses in routine clinical practice.
- Previous studies have largely described overall treatment patterns without distinguishing implementation across healthcare settings or disease stages.

**WHAT THIS STUDY ADDS**

- In two nationwide healthcare systems, GDMT persistence was consistently lower when treatment was initiated in outpatient versus inpatient care.
- Uptake of sodium–glucose cotransporter-2 inhibitors was rapid among patients with newly diagnosed heart failure but remained markedly low among patients with established disease.

**HOW THIS STUDY MIGHT AFFECT RESEARCH, PRACTICE OR POLICY**

- This study encourages the development of systematic treatment optimisation strategies and stronger integration between specialist and primary care to ensure sustained delivery of GDMT for heart failure.

## Introduction

The prevalence of heart failure (HF) is expected to increase beyond the estimated 64 million adults that it currently affects.^1 2^ Sodium–glucose cotransporter-2 (SGLT2) inhibitors were recently recommended as the fourth pillar of guideline-directed medical therapy (GDMT) for HF.^3 4^ In this rapidly evolving treatment landscape, contemporary real-world data are needed to evaluate the utilisation of GDMT and identify potential gaps in the implementation of evidence-based therapies.^4^ This study assessed the implementation of GDMT for HF across Norway and Sweden, focusing on treatment persistence across care settings and the uptake of SGLT2 inhibitors.

## Materials and methods

### Study setting and data sources

This nationwide observational study used data from Patient, Prescribed Drug and Cause of Death Registers in Norway and Sweden (online supplemental methods 1).

### Patient and public involvement

Patients and the public were not involved in the design, conduct, reporting, or dissemination plans of this study.

### Study population

Patients aged ≥18 years were included if they had a recent registered diagnosis of HF, in any position, in *outpatient specialist* or *inpatient hospital care* and who then initiated GDMT (new users) for HF between 1 January 2022 and 31 December 2022. Patients were considered new users of a given GDMT for HF if there was no record of dispensation for any drug in the same GDMT class in the 12 months prior to the index date. The index date was defined as the date of any new GDMT initiation. Baseline characteristics and comorbidities were described at index, and drug treatment was described within the year prior to index (online supplemental methods 2).

### Utilisation of guideline-directed medical therapy for heart failure

Initiation of GDMT for HF was defined by dispensed prescriptions for SGLT2 inhibitors (dapagliflozin or empagliflozin), mineralocorticoid receptor antagonists (MRAs), angiotensin receptor-neprilysin inhibitors sacubitril/valsartan (ARNIs), renin-angiotensin system inhibitors (RASi) and/or beta blockers (online supplemental methods 3). In Norway and Sweden, dapagliflozin and empagliflozin were approved for HF with reduced ejection fraction (HFrEF) in quarter four of 2020 and for HF with preserved ejection fraction (HfpEF) in quarter one of 2022.

### Healthcare level of newly initiated treatment

Treatment initiation was classified according to the timing of the first outpatient pharmacy dispensation relative to hospital discharge. Given that dispensed drug registries capture outpatient dispensations only and do not include medications prescribed or dispensed during hospitalisation, a 14-day postdischarge window was used to identify treatment initiated during the acute postdischarge implementation phase (online supplemental methods 4).

Accordingly, if a GDMT for HF was dispensed within 14 days of a HF diagnosis in *outpatient care*, it was assumed that treatment was initiated in *outpatient care*. If a GDMT was dispensed within 14 days of a HF diagnosis in *inpatient care*, it was assumed that treatment was initiated in *inpatient care*. However, if dispensed >14 days after a HF diagnosis, it was assumed that treatment was initiated in *outpatient care*, regardless of whether the HF diagnosis was registered in *outpatient* or *inpatient care*.

#### Analysis of drug utilisation

Titration and discontinuation of GDMT for HF was followed in patients for up to 12 months from treatment initiation, up until 31 December 2022 or death, whichever occurred first. If a patient’s drug supply finished, treatment was considered discontinued until a new prescription was filled. The target dose for each drug was defined according to the latest guidelines.^3 4^ Drug doses were categorised as a starting dose, low dose (<50% of the target dose), intermediate dose (50–99% of the target dose) or the target dose. Given that initiation, persistence and target-dose attainment were evaluated separately for each GDMT class, patients could contribute to more than one treatment-specific analysis if they initiated multiple therapies during follow-up.

### Uptake of SGLT2 inhibitors

The uptake of SGLT2 inhibitors between 1 January 2020 and 31 December 2023 was assessed in patients with *incident HF* and in patients with *prevalent HF*. The proportion of patients treated with SGLT2 inhibitors was described cross-sectionally and per quarter.

Patients with *incident HF* included those who initiated dapagliflozin or empagliflozin treatment within 3 months of their first ever outpatient or inpatient visit where HF was the main diagnosis. The proportions of patients with *incident HF* who initiated within 3 months was calculated for each quarter between the beginning of 2020 and the end of 2023. This definition was like that used by the national HF registries (the Norwegian Hjertesviktsregisteret and the Swedish RiskSvikt) where patients are actively registered following a hospital visit for HF.

The uptake of SGLT2 inhibitors for the treatment of patients with *prevalent HF* was also assessed each quarter during the same time period. For a patient to be included in a specific quarter, they had to have recorded an *outpatient* or *inpatient care* visit where HF was the main diagnosis any time prior to the quarter of interest; and they had to be alive throughout the entirety of that quarter. The proportion of patients who filled a prescription for dapagliflozin or empagliflozin during that quarter of interest was calculated.

## Results

During 2022, a total of 112 018 patients initiated GDMT for HF across Norway and Sweden, including 57 779 patients who initiated treatment in *outpatient specialist care* and 54 239 who initiated treatment in *inpatient hospital care* (table 1). Baseline characteristics were broadly similar between the two countries and across care settings (online supplemental tables 1 and 2).

**Table 1:** Pooled characteristics of patients from Norway and Sweden who initiated GDMT for heart failure in *outpatient* or *inpatient specialist care*

	*GDMT initiated in outpatient care*						*GDMT initiated in inpatient care*					
	**SGLT2i**	**MRA**	**ARNI**	**ARB**	**ACEi**	**BB**	**SGLT2i**	**MRA**	**ARNI**	**ARB**	**ACEi**	**BB**
*Number of patients*	31 893	26 117	10 731	3582	2801	4248	22 647	30 244	6839	4882	9395	12 896
*Age, years, mean (SD)*	74 (12)	75 (12)	70 (12)	75 (13)	75 (14)	74 (14)	75 (12)	76 (12)	70 (13)	75 (14)	72 (14)	72 (14)
*Female, n (%)*	10 221 (32)	9818 (38)	2548 (24)	1474 (41)	1066 (38)	1569 (37)	8070 (36)	12 561 (42)	1787 (26)	1983 (41)	3391 (36)	4673 (36)
*Comorbidities, n (%)*												
*Ischaemic heart disease*	7491 (23)	5556 (21)	3025 (28)	674 (19)	643 (23)	986 (23)	7192 (32)	8493 (28)	2304 (34)	1465 (30)	3618 (39)	4434 (34)
*Chronic kidney disease*	5106 (16)	3966 (15)	1304 (12)	826 (23)	575 (21)	999 (24)	4373 (19)	4943 (16)	1182 (17)	872 (18)	1172 (12)	1933 (15)
*Diabetes*	7716 (24)	5673 (22)	2612 (24)	758 (21)	501 (18)	815 (19)	5945 (26)	7123 (24)	1866 (27)	779 (16)	1289 (14)	2219 (17)
*Atrial fibrillation*	16 201 (51)	13 570 (52)	4758 (44)	1883 (53)	1382 (49)	1954 (46)	12 315 (54)	16 618 (55)	3305 (48)	2356 (48)	4083 (43)	6006 (47)
*Stroke*	1222 (4)	1167 (4)	429 (4)	175 (5)	168 (6)	307 (7)	1020 (5)	1431 (5)	293 (4)	248 (5)	409 (4)	594 (5)
*PAD*	946 (3)	776 (3)	301 (3)	125 (3)	102 (4)	165 (4)	779 (3)	1029 (3)	299 (4)	132 (3)	221 (2)	300 (2)
*Cancer*	3628 (11)	3320 (13)	1048 (10)	482 (13)	384 (14)	636 (15)	2404 (11)	3369 (11)	637 (9)	561 (11)	973 (10)	1345 (10)
*GDMT, n (%)*												
*SGLT2i*	0 (0)	10 758 (41)	6381 (59)	958 (27)	754 (27)	1029 (24)	0 (0)	12 527 (41)	4407 (64)	1399 (29)	3062 (33)	4077 (32)
*MRA*	19 778 (62)	0 (0	7661 (71)	1252 (35)	916 (33)	1371 (32)	14 307 (63)	0 (0)	4928 (72)	2013 (41)	3554 (38)	4978 (39)
*ARNI*	8209 (26)	4017 (15)	0 (0)	363 (10)	111 (4)	327 (8)	4037 (18)	3525 (12)	0 (0)	121 (2)	94 (1)	1083 (8)
*RASi*	25 084 (79)	21 569 (83)	9914 (92)	0 (0)	0 (0)	3054 (72)	18 807 (83)	24 750 (82)	4673 (68)	0 (0)	0 (0)	10 420 (81)
*BB*	29 441 (92)	23 549 (90)	10 228 (95)	2957 (83)	2329 (83)	0 (0)	20 910 (92)	27 109 (90)	6558 (96)	4299 (88)	8367 (89)	0 (0)
*Other treatment, n (%)*												
*Loop diuretics*	20 275 (64)	17 545 (67)	6580 (61)	2234 (62)	1689 (60)	2424 (57)	15 906 (70)	22 484 (74)	4795 (70)	3105 (64)	5238 (56)	7259 (56)
*Ivabradine*	147 (0)	67 (0)	66 (1)	15 (0)	<5 (0)	8 (0)	34 (0)	23 (0)	22 (0)	<5 (0)	7 (0)	13 (0)
*Nitrates*	6308 (20)	5040 (19)	2242 (21)	656 (18)	471 (17)	649 (15)	5099 (23)	6252 (21)	1529 (22)	1122 (23)	1933 (21)	2252 (17)
*Vitamin K antagonists*	3437 (11)	2665 (10)	1213 (11)	392 (11)	244 (9)	347 (8)	2053 (9)	2770 (9)	738 (11)	370 (8)	625 (7)	789 (6)
Receptor P2Y12 antagonists	4476 (14)	3584 (14)	1962 (18)	366 (10)	364 (13)	574 (14)	4657 (21)	5540 (18)	1508 (22)	1025 (21)	2874 (31)	3541 (27)

* Values of 0 (0) reflect the new-user design, whereby patients initiating a given GDMT class had no prior use of that same class in the 12 months before index.

ACEi, ACE inhibitor; ARB, angiotensin receptor blocker; ARNI, angiotensin receptor-neprilysin inhibitor; BB, beta-blockers; GDMT, guideline-directed medical therapy; MRA, mineralocorticoid receptor antagonist; PAD, peripheral artery disease; RASi, renin-angiotensin system inhibitor; SGL2i, sodium–glucose cotransporter 2 inhibitor.

### Persistence and dose utilisation of guideline-directed medical therapy for heart failure

Of the 54 540 patients who initiated SGLT2 inhibitor treatment, more than 80% remained on treatment at the target dose by the end of follow-up (figure 1). For patients who initiated MRA (n=56 361), RASi or beta-blocker treatment (n=17 144), the proportions treated with the target dose ranged from <5% for angiotensin receptor blockers (n=8464) and beta-blockers to ~23% for ACE system inhibitors (n=12 196). Less than 5% of patients switched from a RASi to an ARNI. The proportion of patients on ARNI treatment who reached the target dose was almost 40% by the end of follow-up. Discontinuation rates were also low with ARNI treatment, ~10% by end of follow-up. Compared with *inpatient hospital care* initiation, discontinuation rates were slightly higher in patients who initiated GDMT in *outpatient specialist care*. Drug utilisation patterns were strikingly similar across countries (online supplemental figures 1 and 2).

**Figure 1:**
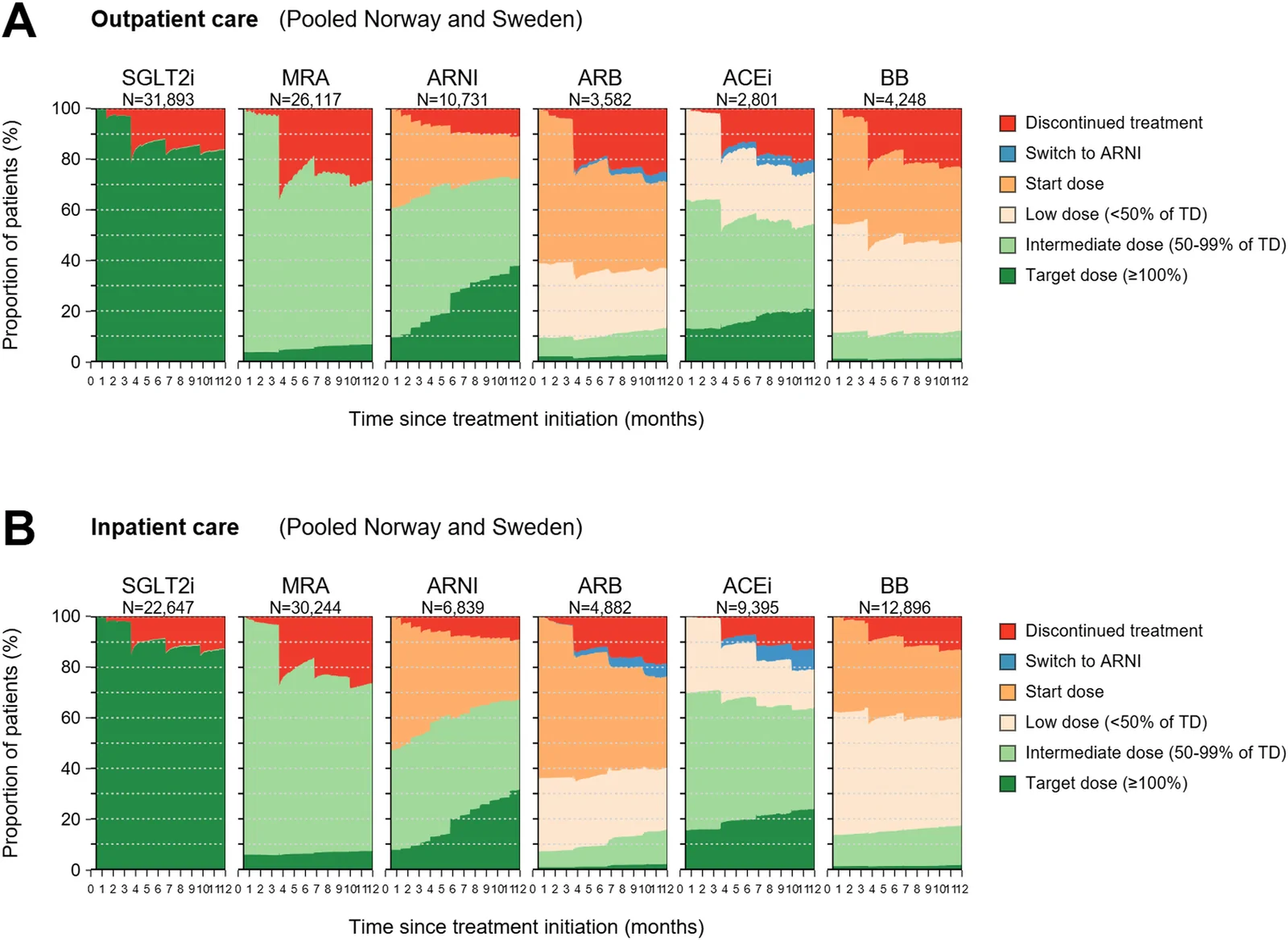
Persistence and dose titration of GDMT following initiation after a recent heart failure hospital visit. Treatment initiation was classified as outpatient or inpatient care according to the definitions in the Methods. Patients were followed for up to 12 months, from treatment initiation to end of 2022 or death; and could have been included in analyses for more than one medication. ACEi, ACE inhibitor; ARB, angiotensin receptor blockers; ARNI, angiotensin receptor-neprilysin inhibitor; BB, beta-blockers; GDMT, guideline-directed medical therapy; MRA, mineralocorticoid receptor antagonist; SGLT2i, sodium-glucose cotransporter-2 inhibitor; TD, target dose.

### Uptake of SGLT2 inhibitors

Uptake of SGLT2 inhibitors increased rapidly among patients with *incident HF* between 2020 and 2023 (figure 2). Uptake after *incident HF* was similar regardless of care setting and consistent between the two countries. The most recent observation shows that approximately 45% of patients in Norway (2022) and 55% of patients in Sweden (2023) received SGLT2 inhibitor therapy within 3 months of a HF hospital visit. In contrast, SGLT2 inhibitor uptake among patients with *prevalent HF* remained substantially lower throughout the study period, reaching only 10–12% by the end of follow-up. Registry data from the Norwegian Hjertesviktregisteret and the Swedish RiksSvikt showed similar patterns of SGLT2 inhibitor uptake.

**Figure 2:**
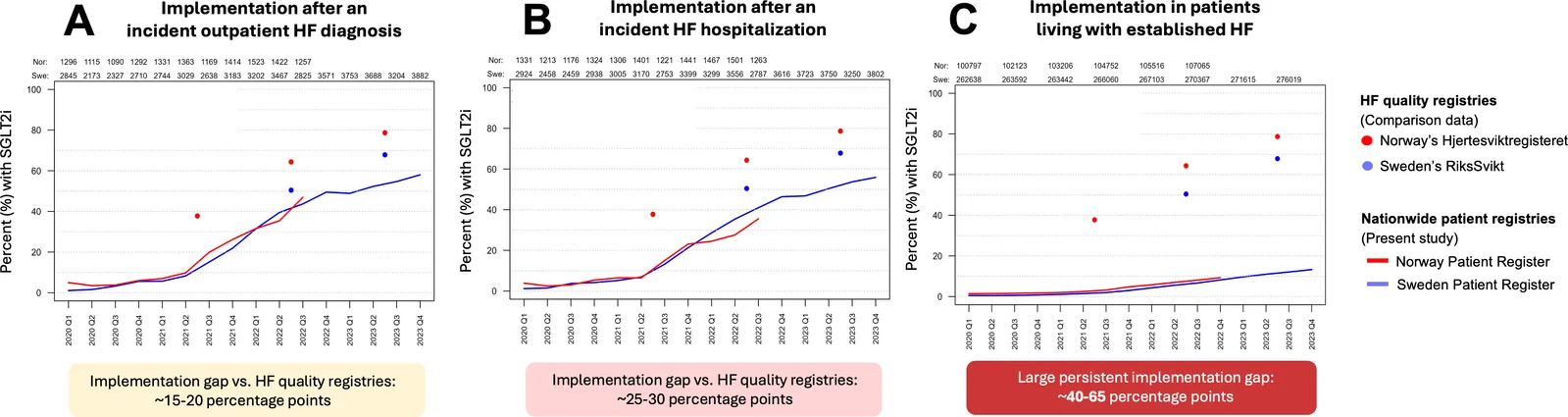
Uptake of SGLT2i after incident heart failure versus among patients with established heart failure in Norway and Sweden (2020–2023). (A) The proportion of patients with a recent incident outpatient diagnosis of heart failure who initiated SGLT2i treatment within 3 months. (B) The corresponding proportion following an incident heart failure hospitalisation. (C) Quarterly cross-sectional SGLT2i use among all patients with established (prevalent) heart failure nationwide. SGLT2i include dapagliflozin and empagliflozin. In Norway and Sweden, SGLT2i were approved for HF with reduced ejection fraction in quarter four of 2020 and for HF with preserved ejection fraction in quarter one of 2022. HF, heart failure; SGLT2i, sodium-glucose cotransporter-2 inhibitor.

## Discussion

In this two-country, nationwide study, which used population-based registry data from Norway and Sweden to evaluate the utilisation of contemporary GDMT for HF across different healthcare settings, two distinct and previously under-recognised implementation gaps emerged. First, persistence with GDMT for HF was consistently lower when treatment was initiated in *outpatient specialist care* compared with that initiated in *inpatient care*. Although differences in overall discontinuation rates were modest, this pattern suggests that initiation of HF therapy in outpatient settings requires close follow-up to be persistent and effective.

Second, there was a marked difference in the uptake of SGLT2 inhibitors between patients with newly diagnosed HF and those with established disease. Indeed, SGLT2 inhibitor uptake increased rapidly between 2020 and 2023 among patients with *incident HF* following a hospital visit. In contrast, utilisation among patients with *prevalent HF* remained substantially lower throughout the study period, suggesting slower implementation of novel therapies in patients already established in chronic HF care.

Additionally, persistence and achievement of target doses differed across GDMT classes. Treatment persistence and target dosing was highest for SGLT2 inhibitor and ARNI treatment, whereas other GDMT classes required more gradual titration and demonstrated higher discontinuation rates.

Together, these findings highlight important structural challenges in the implementation of contemporary HF therapy, even in two Nordic publicly funded, universal healthcare systems with high accessibility to care. The consistency of these findings across two independent nationwide healthcare systems suggests that they are not system-specific but rather reflect broader challenges in the implementation of contemporary HF care.

### Utilisation of guideline-directed medical therapy for heart failure

While GDMT discontinuation rates and target-dose achievement have been described previously,^5 6^ differences according to the healthcare setting in which treatment is initiated have not previously been reported. In the present study, persistence with GDMT was generally higher when treatment was initiated during *inpatient care* compared with *outpatient specialist care*. In addition, a subtle but consistent pattern suggested slightly greater target-dose attainment following *inpatient care* initiation.

The reasons for these differences cannot be fully explained using the available data. However, lower discontinuation rates following postdischarge hospital initiation may reflect characteristics of the immediate postdischarge period rather than the care setting itself. Initiation shortly after hospitalisation occurs during an acute phase of illness, when both clinician and patient awareness of HF and its prognosis are heightened. Treatment decisions are often reinforced through multidisciplinary discharge planning, closer monitoring, medication counselling and early follow-up, which may facilitate treatment acceptance and contribute to improved long-term persistence. In contrast, treatment initiation during routine hospital *outpatient specialist care* may occur later in the disease course, when competing comorbidities, therapeutic inertia and less structured follow-up may contribute to lower treatment persistence.

The inpatient GDMT utilisation patterns observed in the present study, particularly with regard to persistence and dose titration, are broadly consistent with findings from the Swedish EVOLUTION HF study (Utilization of Dapagliflozin and Other Guideline Directed Medical Therapies in Heart Failure Patients: A Multinational Observational Study Based on Secondary Data) covering 2020–2022, and from the multinational analysis by Savarese *et al* (covering 2016–2019).^5 6^ However, inpatient results in the present study appear slightly more favourable compared with earlier EVOLUTION HF data that included patients treated during 2020–2021^6^; potentially suggesting gradual improvements in GDMT utilisation over time, reflected by generally lower discontinuation rates and higher doses. The low discontinuation rates and high target-dose attainment observed for SGLT2 inhibitors are also consistent with results from the EVOLUTION HF study.^6^

The high target-dose attainment observed with SGLT2 inhibitors should be interpreted in the context of the implementation characteristics of this drug class rather than as evidence of superior adherence or prescribing behaviour. Unlike RASi, beta-blockers and MRA, SGLT2 inhibitors are initiated at a single recommended therapeutic dose without the need for dose titration. In addition, their once-daily administration, broad indication across the HF spectrum and generally favourable safety and tolerability profile facilitate implementation in routine clinical practice. In contrast, other GDMT classes frequently require repeated dose optimisation together with clinical and laboratory monitoring to achieve guideline-recommended target doses, which likely contributes to the lower target-dose attainment observed for these therapies in routine clinical practice.

### Uptake of SGLT2 inhibitors

Among patients with *incident HF*, we observed a rapid uptake of SGLT2 inhibitors between 2020 and 2023, reaching approximately 45–55% across the two countries. These findings mirror those reported from the national HF registries in Norway and Sweden, which have also documented an accelerating uptake of SGLT2 inhibitors over time.^7–9^ However, the uptake observed in the present study was approximately 10–30% lower than that reported by the HF registries, which may reflect the more selected patient populations typically included in registry-based cohorts.

Our results are also broadly consistent with findings from the EVOLUTION HF study, which reported a postdischarge SGLT2 inhibitor uptake of approximately 35% in Sweden by the end of 2022.^9^ In contrast, SGLT2 inhibitor utilisation was substantially lower among the much larger population of patients with *prevalent HF*, reaching only 10–12%; compared with 45–55% among patients with *incident HF* (figure 2).

The marked difference in SGLT2 inhibitor uptake between *incident* and *prevalent HF* represents one of the most important findings of the present study. *Incident HF* cohorts primarily reflect implementation opportunities at the time of diagnosis, whereas *prevalent HF* cohorts reflect the cumulative success of implementing GDMT across the entire population living with HF. A new HF diagnosis or recent hospitalisation provides a major therapeutic opportunity during which contemporary GDMT can be initiated as part of comprehensive specialist assessment, discharge planning and early follow-up. In contrast, optimisation of therapy in patients with established HF relies on repeated outpatient medication review and is more susceptible to therapeutic inertia, fragmented longitudinal care pathways, competing multimorbidity and reduced specialist follow-up.

Our findings are consistent with the recently published multinational CaRe Pathways study, which demonstrated that implementation of GDMT remained suboptimal throughout the longitudinal cardiorenal disease trajectory and increased only modestly following the diagnosis of concomitant HF and chronic kidney disease, despite repeated contacts with primary care physicians and hospital specialists.^10^ These observations suggest that major clinical events create critical opportunities for treatment implementation, whereas optimisation during chronic disease follow-up remains substantially more challenging. Collectively, these findings support the concept that implementation gaps in HF extend beyond individual prescribing decisions and reflect system-level challenges across the continuum of care, from timely diagnosis and treatment initiation to long-term optimisation of GDMT. This interpretation is supported by recent multinational implementation studies highlighting persistent therapeutic inertia across the HF care pathway.^11–13^

Although healthcare funding models, reimbursement policies and clinical practice differ internationally, previous multinational studies have demonstrated remarkably consistent patterns of GDMT implementation across diverse healthcare systems, including both universal healthcare and insurance-based models.^5 6 14^ While absolute treatment uptake varies according to healthcare structures, reimbursement policies and national guideline implementation, persistent implementation gaps have been observed across countries. Notably, although Sweden demonstrated somewhat higher uptake of four-pillar GDMT than the USA, the UK and Japan, substantial implementation gaps remained in all participating countries.^10^ Similar implementation inertia has also been observed in recent nationwide chronic kidney disease studies from Norway and Finland following approval of the chronic kidney disease indication for SGLT2 inhibitors despite universal healthcare access.^13–15^ Collectively, these findings suggest that the implementation barriers identified in the present study are unlikely to be unique to Norway and Sweden, but rather reflect broader challenges in translating guideline recommendations into routine clinical practice.

### Limitations

Although the analyses were based on nationwide registries with complete coverage of filled prescriptions, hospital visits and causes of death, important clinical variables were not available. These included vital signs, laboratory data (such as potassium levels, estimated glomerular filtration rate and natriuretic peptides), and left ventricular ejection fraction.

A lack of left ventricular ejection fraction data precluded differentiation of treatment between patients with HFrEF, HFmrEF (HF with mildly reduced ejection fraction) or HFpEF. Consequently, eligibility for individual GDMT classes could not be determined. This is particularly relevant for analyses of SGLT2 inhibitor uptake, as guideline recommendations expanded from HFrEF to HFpEF during the study period. However, the primary persistence and dose analyses were performed among patients who had already initiated treatment and are therefore less influenced by uncertainty regarding HF phenotype. Previous multinational registry studies using similar data sources have reported comparable limitations while demonstrating substantial opportunities to improve GDMT implementation.^10^

In addition, information on socioeconomic status and reasons for treatment discontinuation, such as adverse effects or tolerability issues, was not available. Data for non-steroidal MRAs were also not included, reflecting their recent approval in Europe and limited reimbursement during the study period in Norway and Sweden. Finally, several of the medications studied are also approved for indications other than HF, which may have influenced treatment patterns observed in this analysis.

As is the nature of observational registry studies, residual confounding cannot be excluded. Although Norway and Sweden have universal healthcare systems, unmeasured differences in provider practices, patient health literacy, treatment preferences and other patient-related factors may have influenced treatment initiation, adherence and persistence. These factors were not captured in the available registries and should be considered when interpreting the findings.

Although some patients may fill hospital-initiated prescriptions more than 14 days after discharge, potentially leading to misclassification as outpatient initiation, such misclassification cannot be excluded and reflects the inherent constraints of prescription registry data, which do not capture inpatient prescribing.

The *prevalent HF* analyses were based on patients alive during each calendar quarter and therefore may be influenced by survivor bias. However, the direction and magnitude of any resulting bias are uncertain, since longer survival provides greater opportunity for treatment initiation, whereas patients with more advanced disease may die before receiving therapy.

Although the present study was not designed to evaluate clinical outcomes, the therapies examined constitute GDMT with well-established prognostic benefits demonstrated in randomised clinical trials. Accordingly, our findings should be interpreted as describing implementation patterns rather than the clinical consequences of those patterns.

## Conclusion

In this nationwide study across two Nordic countries, implementation of contemporary GDMT for HF revealed important gaps in routine clinical practice. While uptake of SGLT2 inhibitors increased rapidly among patients with *incident HF*, utilisation remained substantially lower among the larger population with established HF and treatment persistence was modestly lower when therapy was initiated in *outpatient care*. These findings highlight opportunities to improve optimisation of guideline-directed therapy, particularly in chronic HF management across outpatient and primary care settings.

## Supplementary material

10.1136/openhrt-2026-004245 online supplemental file 1

## Data Availability

Data are available upon reasonable request.

## References

[1] Savarese G, Becher PM, Lund LH (2023). Global burden of heart failure: a comprehensive and updated review of epidemiology. Cardiovasc Res.

[2] Khan MS, Shahid I, Bennis A (2024). Global epidemiology of heart failure. Nat Rev Cardiol.

[3] McDonagh TA, Metra M, Adamo M (2021). ESC Guidelines for the diagnosis and treatment of acute and chronic heart failure: Developed by the Task Force for the diagnosis and treatment of acute and chronic heart failure of the European Society of Cardiology (ESC) With the special contribution of the Heart Failure Association (HFA) of the ESC. Eur Heart J.

[4] McDonagh TA, Metra M, Adamo M (2023). Focused Update of the 2021 ESC Guidelines for the diagnosis and treatment of acute and chronic heart failure: Developed by the task force for the diagnosis and treatment of acute and chronic heart failure of the European Society of Cardiology (ESC) With the special contribution of the Heart Failure Association (HFA) of the ESC. Eur Heart J.

[5] Savarese G, Bodegard J, Norhammar A (2021). Heart failure drug titration, discontinuation, mortality and heart failure hospitalization risk: a multinational observational study (US, UK and Sweden). Eur J Heart Fail.

[6] Savarese G, Kishi T, Vardeny O (2023). Heart Failure Drug Treatment-Inertia, Titration, and Discontinuation: A Multinational Observational Study (EVOLUTION HF). JACC Heart Fail.

[7] (2025). 2024 annual report swedehf.

[8] Lægran M, Slind E, Bach C (2025). Norsk Hjertesviktregister Årsrapport for 2024.

[9] Bozkurt B, Savarese G, Adamsson Eryd S (2023). Mortality, Outcomes, Costs, and Use of Medicines Following a First Heart Failure Hospitalization: EVOLUTION HF. JACC Heart Fail.

[10] Greene SJ, Kondo T, Bodegard J (2026). Cardiorenal Disease Pathways and Treatment of Patients With Comorbid Heart Failure and Chronic Kidney Disease. JACC Heart Fail.

[11] Docherty KF, Heywood B, Bayes-Genis A (2026). Benefit of early initiation of disease-modifying therapy in community-based patients with suspected heart failure. Eur Heart J.

[12] Anderson L, Bayes-Genis A, Bodegård J (2025). Suspected de novo heart failure in outpatient care: the REVOLUTION HF study. Eur Heart J.

[13] Tangri N, Svensson MK, Bodegård J (2024). Mortality, Health Care Burden, and Treatment of CKD: A Multinational, Observational Study (OPTIMISE-CKD). Kidney360.

[14] Jenssen TG, Bodegård J, Sveen KA (2025). Prevalence, outcomes, costs, and treatments of a contemporary population with chronic kidney disease in Norway: a nationwide observational study. BMC Nephrol.

[15] Metsärinne K, Bodegård J, Toppila I (2025). Characteristics, treatment and disease burden among stage 3-4 chronic kidney disease patients with and without type 2 diabetes in Finland during 2016-2022. Nephrol Dial Transplant.

