# Supplementary material for: Implementation gaps in contemporary heart failure therapy: a two-country nationwide analysis
Source: Open Heart. 2026 Oct 16;13(2):e004245. doi: 10.1136/openhrt-2026-004245 (PMC13691833; doi:10.1136/openhrt-2026-004245)
Supplement: Supplementary file 1 [file openhrt-13-2-s001.docx]

Supplementary Material

Contents

[Supplementary Methods 1	3](#_Toc234275496)

[Supplementary Methods 2	4](#_Toc234275497)

[Supplementary Methods 3	5](#_Toc234275498)

[Supplementary Methods 4	6](#_Toc234275499)

[Supplementary Tables	7](#_Toc234275500)

[Supplementary Table 1 (Norway).	7](#_Toc234275501)

[Supplementary Table 2 (Sweden).	8](#_Toc234275502)

[Supplementary Figure 1 (Norway)	9](#_Toc234275503)

[Supplementary Figure 2 (Sweden)	10](#_Toc234275505)

[References	11](#_Toc234275507)

Supplementary Methods 1

This study was set in Norway and Sweden, both of which have comprehensive, nationwide, public healthcare systems that residents can access with a minor co-payment for healthcare visits, hospitalisations, and the filling of drug prescriptions.^1^ When a patient accesses the healthcare system, it is mandatory that they report their unique personal identification number, providing a basis for complete population-wide medical history.

The Norwegian Patient Register contains records of all diagnoses and surgical procedures made during visits to outpatient and inpatient clinics in Norway since 2008, with diagnoses recorded according to the International Classification of Disease-10 (ICD-10) coding system ^2^, and surgical procedures recorded according to the Nordic Medico-Statistical Committee Classification of Surgical Procedure system ^3^. The reporting of the cause of death is mandatory in Norway and all deaths that occurred since 1958 are recorded in Norway’s Cause of Death Register according to the ICD-10 system. The Norwegian Prescription Database contains records of all filled prescriptions in Norway, with the type of medication that was dispensed recorded according to the Anatomical Therapeutic Chemical (ATC) Classification system ^4^.

In Sweden, the National Patient Registry has record of all outpatient clinic visit diagnoses from 2001 to present and all hospital discharge diagnoses from 1987 to present. The Prescribed Drug Register has record of all filled drug prescriptions from July 2005 to present. Diagnoses, surgical procedures, cause of death, and medication use are recorded using the same classification systems as used in Norway.

Data were extracted from each country’s Patient Register, Prescription Database, and Cause of Death Register and were linked using the patient’s personal identification number. The resulting databases were managed by Statisticon AB (Uppsala, Sweden).

The study was approved in Norway by the Regional Ethics Committee, Helse Sør-Øst (approval numbers 2015/1337/REK Sør-Øst A and 11744), and the Norwegian Data Inspectorate (Datatilsynet); and in Sweden by the Stockholm Regional Ethics Committee (reference numbers 2021-05240 and 2013/2206-31).

# Supplementary Methods 2

All diagnoses in any position in outpatient care and/or in inpatient care recorded on and prior to the index date were used to identify comorbidities. Use of medications were searched for during the year prior to the index date. Key adverse outcomes including hospitalisations for heart failure, chronic kidney disease, myocardial infarction, stroke, and/or peripheral artery disease were monitored in patients for up to 12 months from the index date until December 31^st^, 2022, or their death, whichever occurred first. Patients with heart failure were identified using ICD-10 codes, I50, I11.0, I13.0, and I13.2.

The following ICD-10 and procedure codes were used to identify comorbidities in patients with a registered diagnosis of heart failure:

|  | **ICD-10 code** | **ATC-code** |
| --- | --- | --- |
| Ischemic heart disease | I20-I25 |  |
| Kidney disease | E10.2, E11.2, E12.2, E13.2, E14.2, N08.3, N17-N19, I12.0, I12.9, I13.1, I13.9, Z49.1, Z49.2, Z99.2 |  |
| Diabetes |  | A10 within 12-months of index; except for any SGLT-2 inhibitor |
| Stroke | I60-I66, G45 |  |
| Atrial fibrillation | I48 |  |
| Peripheral artery disease | I70.2, I73.9, I74.2-9 |  |
| Cancer | C00-C99 |  |

The following ATC codes were used to identify use of medications of interest by each patient with a registered diagnosis of chronic kidney disease, with or without type 2 diabetes:

| **Medication** | **ATC codes** |
| --- | --- |
| SGLT-2 inhibitors | A10BK, A10BD15, A10BD16, A10BD20 |
| Dapagliflozin | A10BK01, A10BD15 |
| RAS inhibitors (ACEi/ARB/ARNi) | C09A, C09B, C09C, C09D (excluding C09DX04) |
| MRA | C03DA |
| Statins | C10AA |
| Loop-diuretics | C03C |
| Low-dose aspirin | B01AC06 |
| Potassium binders | V03AE01, V03AE09, V03AE10 |
| Metformin | A10BA |
| Sulphonylureas | A10BB |
| DPP-4 inhibitors | A10BH |
| GLP-1 agonists | A10BJ |
| Insulin | A10A |

SGLT-2, sodium-glucose cotransporter 2; RAS denotes renin-angiotensin system; ACEi, angiotensin-converting enzyme inhibitors; ARB, angiotensin receptor blockers; ARNi, angiotensin receptor-neprilysin inhibitors; DPP-4, dipeptidyl peptidase-4; glucagon-like peptide-1.

# Supplementary Methods 3

The following ATC codes were used to identify use of guideline-directed medical therapies for heart failure:

| **Medication** | **ATC codes** |
| --- | --- |
| SGLT-2 inhibitors |  |
| Dapagliflozin | A10BK01, A10BD15 |
| Empagliflozin | A10BK03 |
| MRA | C03DA |
| ARNI | C09DX04 |
| RAS inhibitors (ARB/ACEi) | C09A, C09B, C09C, C09D (excluding C09DX04) |
| Beta blockers | C07 |

SGLT-2, sodium-glucose cotransporter 2; MRA, mineralocorticoid receptor antagonists; ARNI, angiotensin receptor-neprilysin inhibitors; RAS, renin-angiotensin system; ARB, angiotensin receptor blockers; ACEi, angiotensin-converting enzyme inhibitors.

# Supplementary Methods 4

Treatment initiation was classified according to the timing of the first outpatient drug dispensation relative to hospital discharge. The nationwide dispensed drug registries capture medications dispensed by community pharmacies, including prescriptions issued at hospital discharge, but do not capture medications administered or supplied for use during hospitalization. In routine clinical practice, patients are commonly discharged with a temporary ("bridge") supply of medication, typically sufficient for several days, allowing time to obtain their first outpatient pharmacy dispensation. Consequently, in-hospital-initiated treatment may first become observable in the dispensed drug registries several days after discharge. A 14-day post-discharge window was therefore selected as a pragmatic approach to capture treatment initiated during the acute post-discharge implementation phase while minimizing misclassification as routine hospital outpatient specialist care. Similar pragmatic post-discharge attribution windows have been applied in previous multinational heart failure registry studies using similar registry infrastructure, with the duration adapted to national dispensing practices.

# Supplementary Tables

Supplementary Table 1 (Norway). Characteristics of patients in Norway who initiated guideline-directed medical therapy for heart failure in outpatient or inpatient specialist care

|  | **GDMT initiated in outpatient specialist care** | | | | | | **GDMT initiated in inpatient specialist care** | | | | | |
| --- | --- | --- | --- | --- | --- | --- | --- | --- | --- | --- | --- | --- |
|  | **SGLT2i** | **MRA** | **ARNI** | **ARB** | **ACEi** | **BB** | **SGLT2i** | **MRA** | **ARNI** | **ARB** | **ACEi** | **BB** |
| **N** | 5,648 | 5,140 | 2,680 | 172 | 714 | 1,048 | 3,976 | 5,569 | 1,838 | 323 | 3,566 | 4,785 |
| **Age, years, mean (SD)** | 70 (12) | 73 (12) | 68 (12) | 74 (14) | 78 (12) | 77 (13) | 71 (12) | 73 (13) | 69 (12) | 73 (14) | 70 (14) | 70 (14) |
| **Female** | 1438 (25) | 1709 (33) | 600 (22) | 68 (40) | 307 (43) | 444 (42) | 1060 (27) | 1979 (36) | 438 (24) | 130 (40) | 1201 (34) | 1648 (34) |
| **Comorbidities, N (%)** |  |  |  |  |  |  |  |  |  |  |  |  |
| Ischemic heart disease | 1362 (24) | 1086 (21) | 746 (28) | 29 (17) | 197 (28) | 302 (29) | 1285 (32) | 1521 (27) | 567 (31) | 74 (23) | 1432 (40) | 1799 (38) |
| CKD | 883 (16) | 853 (17) | 360 (13) | 78 (45) | 228 (32) | 324 (31) | 935 (24) | 1202 (22) | 424 (23) | 98 (30) | 547 (15) | 808 (17) |
| Diabetes | 1282 (23) | 1010 (20) | 544 (20) | 34 (20) | 128 (18) | 172 (16) | 1117 (28) | 1192 (21) | 465 (25) | 57 (18) | 496 (14) | 786 (16) |
| Atrial fibrillation | 2219 (39) | 2304 (45) | 1024 (38) | 80 (47) | 359 (50) | 501 (48) | 1971 (50) | 2885 (52) | 872 (47) | 139 (43) | 1538 (43) | 2130 (45) |
| Stroke total | 195 (3) | 212 (4) | 97 (4) | 13 (8) | 61 (9) | 105 (10) | 188 (5) | 249 (4) | 95 (5) | 17 (5) | 156 (4) | 213 (4) |
| PAD | 189 (3) | 162 (3) | 92 (3) | 8 (5) | 42 (6) | 47 (4) | 136 (3) | 204 (4) | 87 (5) | 13 (4) | 82 (2) | 98 (2) |
| Cancer | 674 (12) | 700 (14) | 287 (11) | 25 (15) | 95 (13) | 174 (17) | 442 (11) | 688 (12) | 215 (12) | 55 (17) | 406 (11) | 552 (12) |
| **GDMT, N (%)** |  |  |  |  |  |  |  |  |  |  |  |  |
| SGLT2i | 0 (0) | 1904 (37) | 1377 (51) | 24 (14) | 105 (15) | 145 (14) | 0 (0) | 2208 (40) | 1056 (57) | 44 (14) | 965 (27) | 1267 (26) |
| MRA | 3185 (56) | 0 (0) | 1499 (56) | 38 (22) | 191 (27) | 263 (25) | 2312 (58) | 0 (0) | 1226 (67) | 91 (28) | 1047 (29) | 1481 (31) |
| ARNI | 2282 (40) | 1254 (24) | 0 (0) | 14 (8) | 35 (5) | 72 (7) | 1069 (27) | 999 (18) | 0 (0) | 7 (2) | 36 (1) | 410 (9) |
| RASi | 4338 (77) | 4133 (80) | 2512 (94) | 0 (0) | 0 (0) | 744 (71) | 3279 (82) | 4498 (81) | 1318 (72) | 0 (0) | 0 (0) | 3880 (81) |
| BB | 5294 (94) | 4627 (90) | 2537 (95) | 135 (78) | 589 (82) | 0 (0) | 3683 (93) | 4955 (89) | 1766 (96) | 260 (80) | 3137 (88) | 0 (0) |
| **Other HF treatment, N (%)** |  |  |  |  |  |  |  |  |  |  |  |  |
| High ceiling diuretics | 3390 (60) | 3391 (66) | 1530 (57) | 127 (74) | 508 (71) | 670 (64) | 2669 (67) | 3957 (71) | 1206 (66) | 218 (67) | 1939 (54) | 2604 (54) |
| Ivabradine | 29 (1) | 8 (0) | 7 (0) | 1 (1) | 0 (0) | 3 (0) | 7 (0) | 2 (0) | 3 (0) | 0 (0) | 1 (0) | 2 (0) |
| Nitrates | 794 (14) | 713 (14) | 373 (14) | 23 (13) | 105 (15) | 129 (12) | 594 (15) | 753 (14) | 282 (15) | 40 (12) | 381 (11) | 390 (8) |
| Vitamin K antagonists | 628 (11) | 548 (11) | 319 (12) | 15 (9) | 67 (9) | 98 (9) | 437 (11) | 543 (10) | 232 (13) | 26 (8) | 278 (8) | 381 (8) |
| Receptor P2Y12 antagonists | 1096 (19) | 880 (17) | 615 (23) | 29 (17) | 126 (18) | 210 (20) | 1090 (27) | 1290 (23) | 477 (26) | 55 (17) | 1234 (35) | 1545 (32) |
| Pacemaker or defibrillator | 73 (1) | 51 (1) | 20 (1) | 1 (1) | 3 (0) | 9 (1) | 51 (1) | 47 (1) | 25 (1) | 0 (0) | 14 (0) | 11 (0) |

Patients could have been could have been included in more than one medication group. GDMT, guideline-directed medical therapy; SGLT2i, sodium-glucose cotransporter 2 inhibitors; MRA, mineralocorticoid receptor antagonists; ARNI, angiotensin receptor-neprilysin inhibitor; ARB, angiotensin receptor blockers; ACEi, angiotensin-converting enzyme inhibitors; SD, standard deviation; CKD, chronic kidney disease; PAD; peripheral artery disease; RAASi, renin-angiotensin aldosterone system inhibitors; HF, heart failure.

Supplementary Table 2 (Sweden). Characteristics of patients in Sweden who initiated guideline-directed medical therapy for heart failure in outpatient or inpatient specialist care

|  | **GDMT initiated in outpatient specialist care** | | | | | | **GDMT initiated in inpatient specialist care** | | | | | |
| --- | --- | --- | --- | --- | --- | --- | --- | --- | --- | --- | --- | --- |
|  | **SGLT2i** | **MRA** | **ARNI** | **ARB** | **ACEi** | **Betablockers** | **SGLT2i** | **MRA** | **ARNI** | **ARB** | **ACEi** | **Betablockers** |
| **N** | 26,245 | 20,977 | 8,051 | 3,410 | 2,087 | 3,200 | 18,671 | 24,675 | 5,001 | 4,559 | 5,829 | 8,111 |
| **Age, years, mean (SD)** | 74 (11) | 75 (12) | 70 (12) | 75 (13) | 74 (14) | 74 (14) | 76 (12) | 77 (12) | 70 (13) | 75 (14) | 73 (14) | 73 (14) |
| **Female** | 8783 (33) | 8109 (39) | 1948 (24) | 1406 (41) | 759 (36) | 1125 (35) | 7010 (38) | 10582 (43) | 1349 (27) | 1853 (41) | 2190 (38) | 3025 (37) |
| **Comorbidities, N (%)** |  |  |  |  |  |  |  |  |  |  |  |  |
| Ischemic heart disease | 6129 (23) | 4470 (21) | 2279 (28) | 645 (19) | 446 (21) | 684 (21) | 5907 (32) | 6972 (28) | 1737 (35) | 1391 (31) | 2186 (38) | 2635 (32) |
| CKD | 4223 (16) | 3113 (15) | 944 (12) | 748 (22) | 347 (17) | 675 (21) | 3438 (18) | 3741 (15) | 758 (15) | 774 (17) | 625 (11) | 1125 (14) |
| Diabetes | 6434 (25) | 4663 (22) | 2068 (26) | 724 (21) | 373 (18) | 643 (20) | 4828 (26) | 5931 (24) | 1401 (28) | 722 (16) | 793 (14) | 1433 (18) |
| Atrial fibrillation | 13982 (53) | 11266 (54) | 3734 (46) | 1803 (53) | 1023 (49) | 1453 (45) | 10344 (55) | 13733 (56) | 2433 (49) | 2217 (49) | 2545 (44) | 3876 (48) |
| Stroke total | 1027 (4) | 955 (5) | 332 (4) | 162 (5) | 107 (5) | 202 (6) | 832 (4) | 1182 (5) | 198 (4) | 231 (5) | 253 (4) | 381 (5) |
| PAD | 757 (3) | 614 (3) | 209 (3) | 117 (3) | 60 (3) | 118 (4) | 643 (3) | 825 (3) | 212 (4) | 119 (3) | 139 (2) | 202 (2) |
| Cancer | 2954 (11) | 2620 (12) | 761 (9) | 457 (13) | 289 (14) | 462 (14) | 1962 (11) | 2681 (11) | 422 (8) | 506 (11) | 567 (10) | 793 (10) |
| **GDMT, N (%)** |  |  |  |  |  |  |  |  |  |  |  |  |
| SGLT2i | 0 (0) | 8854 (42) | 5004 (62) | 934 (27) | 649 (31) | 884 (28) | 0 (0) | 10319 (42) | 3351 (67) | 1355 (30) | 2097 (36) | 2810 (35) |
| MRA | 16593 (63) | 0 (0) | 6162 (77) | 1214 (36) | 725 (35) | 1108 (35) | 11995 (64) | 0 (0) | 3702 (74) | 1922 (42) | 2507 (43) | 3497 (43) |
| ARNI | 5927 (23) | 2763 (13) | 0 (0) | 349 (10) | 76 (4) | 255 (8) | 2968 (16) | 2526 (10) | 0 (0) | 114 (3) | 58 (1) | 673 (8) |
| RASi | 20746 (79) | 17436 (83) | 7402 (92) | 0 (0) | 0 (0) | 2310 (72) | 15528 (83) | 20252 (82) | 3355 (67) | 0 (0) | 0 (0) | 6540 (81) |
| BB | 24147 (92) | 18922 (90) | 7691 (96) | 2822 (83) | 1740 (83) | 0 (0) | 17227 (92) | 22154 (90) | 4792 (96) | 4039 (89) | 5230 (90) | 0 (0) |
| **Other HF treatment, N (%)** |  |  |  |  |  |  |  |  |  |  |  |  |
| High ceiling diuretics | 16885 (64) | 14154 (67) | 5050 (63) | 2107 (62) | 1181 (57) | 1754 (55) | 13237 (71) | 18527 (75) | 3589 (72) | 2887 (63) | 3299 (57) | 4655 (57) |
| Ivabradine | 118 (0) | 59 (0) | 59 (1) | 14 (0) | 2 (0) | 5 (0) | 27 (0) | 21 (0) | 19 (0) | 3 (0) | 6 (0) | 11 (0) |
| Nitrates | 5514 (21) | 4327 (21) | 1869 (23) | 633 (19) | 366 (18) | 520 (16) | 4505 (24) | 5499 (22) | 1247 (25) | 1082 (24) | 1552 (27) | 1862 (23) |
| Vitamin K antagonists | 2809 (11) | 2117 (10) | 894 (11) | 377 (11) | 177 (8) | 249 (8) | 1616 (9) | 2227 (9) | 506 (10) | 344 (8) | 347 (6) | 408 (5) |
| Receptor P2Y12 antagonists | 3380 (13) | 2704 (13) | 1347 (17) | 337 (10) | 238 (11) | 364 (11) | 3567 (19) | 4250 (17) | 1031 (21) | 970 (21) | 1640 (28) | 1996 (25) |
| Pacemaker or defibrillator | 1558 (6) | 942 (4) | 617 (8) | 143 (4) | 93 (4) | 228 (7) | 1111 (6) | 1196 (5) | 451 (9) | 199 (4) | 230 (4) | 390 (5) |

Patients could have been could have been included in more than one medication group. GDMT, guideline-directed medical therapy; SGLT2i, sodium-glucose cotransporter 2 inhibitors; MRA, mineralocorticoid receptor antagonists; ARNI, angiotensin receptor-neprilysin inhibitor; ARB, angiotensin receptor blockers; ACEi, angiotensin-converting enzyme inhibitors; SD, standard deviation; CKD, chronic kidney disease; PAD; peripheral artery disease; RAASi, renin-angiotensin aldosterone system inhibitors; HF, heart failure.

Supplementary Figure 1 (Norway)

Titration and discontinuation of guideline-directed medical therapy (GDMT) for patients with heart failure in Norway. Patients were followed for up to 12 months, from treatment initiation to end of 2022 or death; and could have been included in analyses for more than one medication. SGLT2i, sodium-glucose cotransporter-2 inhibitors; MRA, mineralocorticoid receptor antagonists; ARNI, angiotensin receptor-neprilysin inhibitors; ARB, angiotensin receptor blockers; ACEi, angiotensin-converting enzyme inhibitors; BB, betablockers.

Supplementary Figure 2 (Sweden)

Titration and discontinuation of guideline-directed medical therapy (GDMT) for patients with heart failure in Sweden. Patients were followed for up to 12 months, from treatment initiation to end of 2022 or death; and could have been included in analyses for more than one medication. SGLT2i, sodium-glucose cotransporter-2 inhibitors; MRA, mineralocorticoid receptor antagonists; ARNI, angiotensin receptor-neprilysin inhibitors; ARB, angiotensin receptor blockers; ACEi, angiotensin-converting enzyme inhibitors; BB, betablockers.

# References

1.	Ludvigsson JF, Bergman D, Lundgren CI, Sundquist K, Geijerstam J-L af, Glenngård AH, et al. The healthcare system in Sweden. *Eur J Epidemiol* 2025;**40**:563–579.

2.	WHO. Classifications. Available from: http://www.who.int/classifications/icd/en/

3.	(NOMESCO) NM-SC. NOMESCO Classification of Surgical Procedures. Available from: https://norden.diva-portal.org/smash/get/diva2:970547/FULLTEXT01.pdf

4.	WHO Collaborating Centre for Drug Statistics Methodology. ATC/DDD Index 2017. Available from: https://www.whocc.no/
